# Neck pain and postural balance among workers with high postural demands - a cross-sectional study

**DOI:** 10.1186/1471-2474-12-176

**Published:** 2011-08-01

**Authors:** Marie B Jørgensen, Jørgen H Skotte, Andreas Holtermann, Gisela Sjøgaard, Nicolas C Petersen, Karen Søgaard

**Affiliations:** 1 National Research Centre for the Working Environment, Copenhagen, Denmark; 2 Department of Physical Exercise and Sport Sciences, The Panum Institute, University of Copenhagen, Copenhagen, Denmark; 3 Institute of Sports Science and Clinical Biomechanics, University of Southern Denmark, Denmark; 4 Department of Neuroscience and Pharmacology, University of Copenhagen

## Abstract

**Background:**

Neck pain is related to impaired postural balance among patients and is highly prevalent among workers with high postural demands, for example, cleaners. We therefore hypothesised, that cleaners with neck pain suffer from postural dysfunction. This cross-sectional study tested if cleaners with neck pain have an impaired postural balance compared with cleaners without neck pain.

**Methods:**

Postural balance of 194 cleaners with (n = 85) and without (N = 109) neck pain was studied using three different tests. Success or failure to maintain the standing position for 30 s in unilateral stance was recorded. Participants were asked to stand on a force platform for 30 s in the Romberg position with eyes open and closed. The centre of pressure of the sway was calculated, and separated into a slow (rambling) and fast (trembling) component. Subsequently, the 95% confidence ellipse area (CEA) was calculated. Furthermore a perturbation test was performed.

**Results:**

More cleaners with neck pain (81%) failed the unilateral stance compared with cleaners without neck pain (61%) (p < 0.01). However, the risk of failure in unilateral stance was statistically elevated in cleaners with concurrent neck/low back pain compared to cleaners without neck/low back pain (p < 0.01), whereas pain at only neck or only low back did not increase the risk. Impaired postural balance, measured as CEA (p < 0.01), rambling (p < 0.05) and trembling (p < 0.05) was observed among cleaners with neck pain in comparison with cleaners without neck pain in the Romberg position with eyes closed, but not with eyes open.

**Conclusions:**

Postural balance is impaired among cleaners with neck pain and the current study suggests a particular role of the slow component of postural sway. Furthermore, the unilateral stance test is a simple test to illustrate functional impairment among cleaners with concurrent neck and low back pain.

**Trial registration:**

ISRCTN96241850

## Background

Cleaning is a physically demanding job involving standing, walking and working in awkward postures [1]. Musculoskeletal disorders are highly prevalent among cleaners, the cause of which is considered to be the high exposure to repetitive tasks and awkward postures [1-3]. The prevalence of sick leave and early retirement is high among cleaners and people with physically demanding jobs [4] and has been related to musculoskeletal disorders [5,6]. Apart from the fact that musculoskeletal pain in itself can be disabling, physical dysfunction related to pain could also contribute to sick leave and early retirement. Therefore, reducing both pain and dysfunction related to pain may be an effective prevention and rehabilitation strategy among people with physically demanding jobs. However, whether dysfunction related to pain is evident in such job groups remains to be established.

According to several studies of patients, neck pain may underlie impaired postural balance [7-11]. Altered motor strategies during work tasks after acute, sub-acute and chronic pain have also been reported [12]. However such studies have not been conducted on postural balance. Instead, previous studies on postural balance and neck pain have primarily involved referred patients, whereas consecutive non-disabled workers have not been studied. Therefore, this study tested if cleaners with neck pain are also characterised by postural impairments.

Through analysis of a variety of postural tasks and outcomes, different elements of postural performance can be revealed. In the clinical setting, the one leg stand test is an easy and quick test of balance often used to estimate risk of falling among elderly patients [13]. Impaired proprioception to a large degree can be overcome by visual feedback [14]. Therefore, impaired proprioception is better investigated with vision removed [8,15,16]. Furthermore, separating the ground reaction force data during standardised body positions into slow and fast components may reveal if postural movements primarily originate from peripheral or central mechanisms [17,18]. Finally, introducing an externally generated perturbation can more consistently challenge proprioception and thus the control of postural balance [19]. Thus, these different tests of postural balance may be useful for investigating if differences in postural performance between cleaners with and without neck pain arise from central or peripheral mechanisms.

The primary aim was to investigate if cleaners with neck pain have reduced postural balance in comparison with cleaners without pain in a large cross-sectional sample. The secondary aim was to investigate the possible mechanisms involved in the postural impairments among cleaners with neck pain.

## Methods

The current study is a cross-sectional study and part of the FINALE programme described elsewhere [20]. The study was approved by the local ethics committee (H-C-2007-0033), registered in the database for randomised controlled trails (ISRCTN96241850), and performed according to the Declaration of Helsinki.

### Participants

A thorough description of the participant recruitment is given elsewhere [21], and is briefly described here. Female participants were recruited from 758 (78% female) cleaning employees with at least 20 working hours each week. The 320 female cleaners who gave positive consent to participate were invited to answer a questionnaire and participate in physical testing. Among those invited, 234 women completed both the questionnaires and the physical testing. Subjects were characterised as having neck pain if they reported > 30 days of neck pain during the previous year and as having no pain if they reported < 8 days of neck pain during the previous year. Of the 234 participants, 40 cleaners reporting pain for 8-30 days during the previous year were not included in the analyses of this study. Thus a total of 194 cleaners were included in this study.

### Measurements

#### Pain questionnaire

Questionnaires were administered to obtain information on musculoskeletal pain using the Standardised Nordic Questionnaires for the analysis of musculoskeletal symptoms [22]. The following question was posed: 'How many days have you had trouble in the neck during the last 12 months?' with the response categories of 0 days; 1-7 days; 8-30 days; > 30 days; every day. The same question was posed for low back pain as it was included as a possible confounder in the later analyses.

#### Procedure

The Questionnaires were completed 1-10 days before the trial. Exclusion from the physical tests (only the physical tests, not the trial as a whole) was determined on the day of the trial and occurred if a participant presented with considerable pain, trauma (strain or overload) or was still physically restricted due to a recent trauma in the neck, low back, hip, knee or ankle. No other exclusion criteria were applied.

Three types of 30-second balance tests and a perturbation test were performed in a room away from disturbances. The participants were encouraged to take a break in between tests whenever they felt a decrease in attention to the task. A crew of trained researchers, blinded to the participants' answers to the questionnaires conducted and gave standardised instructions for each test. Participants were instructed to "stand as steadily as possible". Participants stood barefoot on a force platform (AMTI, platform type OR6-7-1000, amplifier type MSA-6). During the balance test, indications of test progression (10s, 20s, end of test) were conveyed verbally to the participant from the researcher. If the participant moved his/her arms or feet from the starting position and/or lost balance, a new trial was commenced.

#### Unilateral stance

A unilateral stance test was performed with eyes open and participants were instructed to look directly ahead at a black spot placed approximately 2 meters from the force platform at eye height. The participants stood on the dominant foot (defined as the foot used for standing while kicking a ball) with the big toe of the non-dominant foot leaning against the medial malleolus of the dominant foot. The dominant foot was placed parallel to the y-axis of the platform. The test was performed for 30 s. Each participant was allowed three trials with loss of balance before the end of the test being classified as failed. Success or failure to complete the unilateral stance test was registered.

#### Romberg test with open and closed eyes

Two tests were performed - with eyes open and closed- in the Romberg position, defined as standing with feet together, heel-to-heel and toe-to-toe [14]. The participants stood with their arms crossed over their chest and their feet parallel to the y-axis of the platform. In the test with eyes open, which was performed to familiarise the participants with the test situation, participants were instructed to look at the black spot. The test was performed for 30 s. The test was then performed three times with eyes closed.

#### Perturbation test

**Figure 1:**
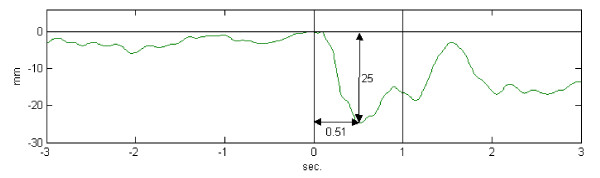
**Anterior-posterior position during the perturbation test**. A record of the anterior-posterior position of the centre of pressure during the perturbation test. The two vertical bars illustrate the 0-1 s time window after the sudden perturbation in which the perturbation occurs. The vertical arrow shows the size of the displacement (mm) and the horizontal arrow the time to peak displacement (s).

The perturbation test was performed with eyes focusing on the black spot, feet in the Romberg position and arms held horizontally forward, a shoulder width apart. The participants held a bar in their hands with a 2.2 kg load fixed to the bar by an electromagnet. The distance from the lateral point of the acromion to the bar was measured. A signal from the computer triggered a release of the load randomly between 5 and 15 seconds after initiation of the test. The release of the load produced a sudden change in external forces acting on the subject, leading to a small anterior-posterior (AP) displacement of the subject's centre of pressure (COP). The perturbation was quantified by the maximal posterior displacement within one second after the drop of the load (Figure 1). The recording ended 2-3 seconds after the load-drop. Three trials were performed. Subjects with severe neck pain or discomfort on the day of testing were excluded from this test.

### Computerised posturography

The force (*Fx, Fy *and *Fz*) and moment (*Mx, My *and *Mz*) signals were sampled at 125 Hz, and filtered (10 Hz 4^th ^order Butterworth zero-phase low-pass filter).

**Figure 2:**
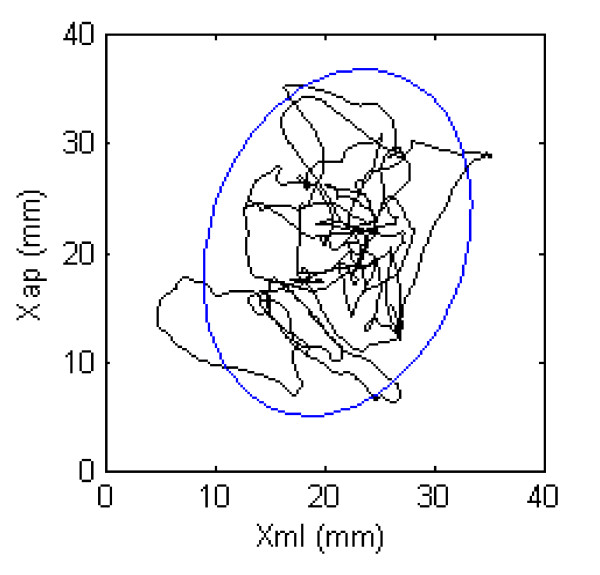
**Ninety-five per cent confidence ellipse area**. Illustration of a cleaners sway area (centre of pressure) during the Romberg with eyes open. The y-axis represents the anterior-posterior axis and the x-axis represents the medio-lateral axis. The blue circle illustrates the 95% confidence ellipse area.

The COP consisting of the AP and medio-lateral (ML) components ([*x_AP_, x_ML_*] = [*Mx/Fz, My/Fz*]) was calculated and separated into a rambling - the slow component of sway - and trembling - fast adjustment based on mechanical properties of muscles and joints, i.e. the myotatic reflex - component [17,23,24]. The instances where *Fx *= 0 and *Fy *= 0 were identified, and the corresponding COP positions determined. These positions were interpolated by a cubic spline procedure to obtain the rambling trajectory and subtracted from the COP trajectory to estimate the trembling component. Subsequently, the 95% confidence ellipse areas were calculated for the COP (COP 95% confidence Ellipse Area, here abbreviated as *CEA*) and for the rambling and trembling components of the COP. The 95% confidence ellipse area is the area of the 95% bivariate ellipse, enclosing approximately 95% of the points on the COP path (Figure 2) [25]. The CEA was calculated as π*a b *where the radii of the ellipse were determined as

where *s_AP _*and *s_ML _*are AP and ML standard deviations, respectively, of the COP data, and *F_.05[2, N-2] _*is the *F *statistic at a 95% confidence level for a bivariate distribution with *N *data points; *F_.05[2, N-2] _*is approximately 3.0 for large sample sizes (*N *> 120). *D *is calculated according to

where *s_APML _*is the covariance

and where  and  are mean values of the COP coordinates.

An example of a 30 s COP path and the corresponding 95% confidence ellipse are shown in Figure 2.

### 2.4. Statistics

For the balance tests with three recorded trials (Romberg with eyes closed and unilateral stance), the outcome variables were calculated for each trial and averaged. Similarly, the average maximal posterior displacement for the three perturbation trials was calculated. All data from the force platform were logarithmically transformed. General linear model (GLM) was used to test for differences among cleaners with and without pain. Covariates of age, height and body weight were included if they were deemed significant. For Romberg tests, three models with stepwise entry of covariates were tested. Model 1 was unadjusted, model 2 was adjusted for significant confounders only and model 3 was adjusted for significant confounders and low back pain. Because several previous studies report a relationship between postural balance and low back pain [26-30], low back pain was introduced as a confounder. For the perturbation test, an unadjusted model was tested and a model was adjusted with significant confounders, arm length and low back pain. The low back pain confounder was based on questionnaire data similar to the ones for neck pain, but applied as a continuous variable (0 days, 1-7 days, 8-30 days, 30-90 days > 90 days or every day). To test differences in failure to perform the unilateral stance between cleaners with and without pain, a chi^2 ^test was conducted. Additionally, the impact on failure frequency was evaluated in groups without neck/low back pain, with neck pain only, with low back pain only and with concurrent neck and low back pain. Binomial logistic regression was performed with failure included as dependent variable and the group of cleaners without pain (neither neck nor low back) was the reference. For that analysis, low back pain was based on questionnaire data dichotomized in the same way as neck pain (> 8 days, < 30 days). Six persons were identified as outliers (> 3 SD from the mean) in the logarithmically transformed trembling data for Romberg with eyes open and removed from further analyses. Similarly, in the logarithmically transformed perturbation data, two outliers (> 3 SD from the mean) were identified and removed. SPSS 17.0 statistical software was used for the statistical analyses. A probability of < 0.05 was considered statistically significant.

## Results

Participants' mean age, height, body weight, and length of employment are shown in Table 1. Body weight was a significant covariate in the statistical analyses for Romberg with eyes open and closed, unilateral stance and the perturbation test and therefore was entered in all adjusted statistical models.

**Table 1:** Baseline characteristics of the participants

	Pain status	N	mean	Range	SD
Age	No pain	109	45	23-69	8.6
(years)	Pain	85	45	24-58	8.2
Height	No pain	109	162	142-180	7.7
(cm)	Pain	85	161	148-180	7.2
Weight	No pain	109	72	44-115	16.1
(kg)	Pain	85	72	49-110	13.2
Length of employment	No pain	88	9	1-27	7.3
(years)	Pain	70	9	0-29	8.4

Age, height, weight and length of employment of cleaners

N = number of participants, SD = standard deviation.

### Unilateral stance test

Eighty-one percent of the cleaners with neck pain did not accomplish the unilateral stance test as opposed to the significantly lower proportion (61%) of the cleaners without neck pain (p = 0.003). Among those accomplishing the test, CEA mean (SD) was 902 (367) and 916 (387) mm^2 ^for the group without and with neck pain, respectively. Due to the skewed failure frequency of the two groups, no further analyses were carried out on the platform data of this test. Failure frequencies, odds ratios and p-values of the groups without neck/low back pain, with neck pain only, with low back pain only and with concurrent neck and low back pain are given in Table 2. The group with concurrent neck and low back pain had a significantly larger failure frequency, than the group without pain, whereas the neck pain only and low back pain only didn't differ from the group without pain.

**Table 2:** Fall frequency at different pain states

Pain status	n	Fall %	OR	95%CI	p
Without pain	44	**56.8**	1.00		
Neck pain	16	**56.3**	0.97	0.3-3.1	0.969
Low back pain	21	**57.1**	1.01	0.4-2.9	0.980
Concurrent	63	**85.7**	4.56	1.8-11.5	0.001

Frequency of falling within the group without pain, with neck pain only, with low back pain only and in the group with concurrent neck and low back pain.

Concurrent = concurrent neck and low back pain corresponding to more than 30 days/year, n = number of participants, OR = odds ratio, CI = confidence intervals, p = level of significance. Significant at the level of p >/= 0.05.

### Romberg with open eyes

In the unadjusted model, no significant difference on CEA among cleaners with and without neck pain was found in the Romberg with eyes open (p = 0.241) (see Table 3). No significant differences in rambling (p = 0.399) or trembling (p = 0.134) were found among cleaners with and without neck pain in the Romberg with eyes open. Neither adjusting for body weight alone, nor both body weight and low back pain materially changed the level of significance.

**Table 3:** The Romberg test and neck pain

		Mean	SD	N	p-value	p-value	p-value
		(mm2)			Model 1	Model 2	Model 3
Romberg open eyes							
CEA	No pain	**470**	286	107	.241	.214	.494
	Pain	**485**	236	84			

Rambling	No pain	**333**	224	107	.399	.375	.627
	Pain	**334**	179	84			

Trembling	No pain	**65**	60	107	.134	.112	.291
	Pain	**67**	51	84			
**Romberg closed eyes**							

CEA	No pain	**699**	386	109	.012*	.009*	.035*
	Pain	**884**	587	85			

Rambling	No pain	**405**	221	109	.012*	.010*	.029*
	Pain	**516**	328	85			

Trembling	No pain	**150**	113	109	.053	.043*	.119
	Pain	**184**	173	85			

Model 1: Unadjusted

Model 2: Adjusted for weight

Model 3: Adjusted for weight and low back pain

* = significant result, CEA = Centre of pressure 95% Confidence Ellipse Area, SD = Standard deviation, Mean, SD and N are given for the unprocessed data.

### Romberg with eyes closed

In the Romberg with eyes closed, cleaners with neck pain had significantly larger CEA than those without neck pain (p = 0.012) (Table 3).

Furthermore, cleaners with neck pain had significantly larger rambling (p = 0.012) than people without neck pain. The significant differences in CEA and rambling remained after adjustment for weight and low back pain. Differences between cleaners with and without neck pain were found for trembling in the weight-adjusted model only (p = 0.043). Adjustment for low back pain modified the difference to an insignificant level (p = 0.119).

### Perturbation test

There was no significant difference among cleaners with neck pain and those without neck pain in the perturbation test (p = 0.938). This result remained after adjustment for arm length, weight and low back pain (p = 0.606).

## Discussion

The main finding of this study was that cleaners with neck pain have an impaired postural balance compared with cleaners without neck pain. This is shown in both the ability to stand on one leg as well as in the Romberg test with eyes closed on CEA, rambling and on trembling.

The Romberg test with eyes closed uncovered a poorer proprioception among cleaners with pain, compared with cleaners without neck pain. Former findings indicate that people with neck pain rely on visual input to compensate for impaired proprioception [8]. Separating the COP into rambling and trembling components also revealed differences between cleaners with and without neck pain. The difference in rambling represents the central component of postural balance [18]. This finding is in accordance with previous reports on neck pain patients [31]. The impaired postural adjustments may arise from the nociceptive input of pain disturbing the proprioceptive information from the muscle spindles in the deep neck muscles, thereby introducing more inaccurate central slow adjustment of balance [16].

A significant difference in trembling between cleaners with and without pain was found. However the difference diminished after adjusting for low back pain. Therefore the relationship between neck pain and trembling seems to be influenced by the presence of low back pain and there is not a strong association regarding trembling and neck pain alone. This finding of no significant difference in trembling between cleaners with and without neck pain is in line with previous studies on neck pain patients, where trembling is not found to be related to neck pain among patients with concurrent low back pain [11]. However, an improvement in trembling has been found in an uncontrolled trial among neck pain patients after a neck coordination training intervention, which also resulted in improved smoothness of muscle activation [32]. This finding indicates potential for improvement of the mechanical muscle properties with coordination training among neck pain patients.

No significant differences in postural performance were observed among cleaners with and without neck pain in the Romberg test with eyes open. This finding may indicate that the cleaners with neck pain can overcome their impaired proprioception by use of their vision. This is supported by previous studies on other populations [14].

The unilateral stance test revealed a large frequency of failure among cleaners with neck pain being significantly different from those without neck pain. However, a large proportion of those with neck pain had concurrent low back pain. The relatively small groups of cleaners with pain in only one of the two sites did not have elevated failure frequencies. A significant and more than four times increased risk of failure during unilateral stance was seen in the group with concurrent neck and low back pain. Previous findings support that particularly concurrence of pain or generalised spinal pain seem to have a role on postural impairments [11]. This finding of a higher frequency of failing to stand on one leg among cleaners with spinal pain compared with cleaners without pain illustrates the functional significance of their postural dysfunction. Note that this test is performed with eyes open, but it seems that this degree of difficulty exceeds the previously proven ability to compensate for the dysfunction in the proprioception by visual input, when the cleaners have pain at two spinal sites. The chosen cut-point of 30 s is a very low demand when for instance compared with community-dwelling elderly women, having a mean standing time of 60 s for the same task [13], and another study of 180 women, where everyone below the age of 50 was able to complete a 30 s stance test. Simple unilateral stance tests with up to 60 s balance have previously been introduced and shown to have good reliability [33] and have been related to back health [34]. The unilateral test could therefore in its very simple execution be used in prospective large-scale studies, for example as a means to reveal if other job groups suffer pain-related sensorimotor dysfunctions. However, a relatively large failure rate was also found among those with neither neck nor low back pain (57-61%). Therefore it is only suitable for evaluations at a group level. Previously, an effect of age has been seen in unilateral tests, and thus the age of those failing could be a possible explanation for the general high failure rate [35]. Among those without neck pain in the current study, no relevant difference in age existed between those failing the test (46 yrs) and those accomplishing the test (44 yrs). Thus the exact explanation for the high failure rate in the current study compared with other studies cannot be explained by age.

### Methodological considerations

This study contributes to previous research by investigating a large sample recruited from a working population. Some weaknesses are present. The results of the CEA from the unilateral stance test are not representative, since the test criteria (i.e. holding balance for 30 s) were above many of the participants' abilities. The test has previously been used on both healthy people and people with work-related neck pain, all being able to complete the test [9]. One reason for the generally poor execution of this test may be that participants with other types of balance disturbances were not specifically excluded from this test. However the participants were all working cleaners and thus severe illness disturbing balance probably does not exist in this group. Nevertheless, the exclusion criteria may not have been sufficient to take out other possible causes of disturbed balance. Thus, different modalities in the execution of the test may explain why other studies have found far better performance in the unilateral test [35]. For example, in the current study we fixated the arms, the non-standing leg and the vision, which may make the test much more difficult to accomplish compared with other modalities. In future research registration of the time to failure would improve the data from unilateral balance tasks and a test of correlations with age and intensity, duration or site of pain could be interesting. Also the results of the perturbation test were not representative among cleaners with pain due to a high level of failure and exclusion. Failure in the perturbation test was primarily due to the cleaners with wrist pain, unable to hold the bar for the duration of the test. Former studies report patients have used it successfully [9], however the reason for the large failure in this study may arise from the fact that cleaners experience pain in several body regions impeding the conduct of the test.

The analysis methods used in the current study have previously proved useful in investigating neck pain and postural balance [9,32].

Finally, due to the cross-sectional design, the current study cannot reveal whether the postural dysfunction is a cause or a consequence of the neck pain. This calls for longitudinal prospective studies in this area.

## Conclusions

Results of the current study indicate that postural imbalance is common among cleaners with neck pain and that neck pain disturbs the central control of postural balance. Furthermore, the simple unilateral stance test revealed significant functional impairments among cleaners with concurrent neck and low back pain. There is a need, though, for randomised controlled trials to establish whether a postural training strategy may reduce neck pain by improving postural deficits or vice versa.

## Competing interests

The authors declare that they have no competing interests.

## Authors' contributions

Design and concept: MBJ, KS, GIS, JHS, and NCP. Conduction: MBJ, KS and AHO, Data processing and statistical analysis: MBJ, JHS. MBJ wrote the first version of the manuscript. All authors took part in interpretation, editing, reading, and approval of the final version of the paper.

## Pre-publication history

The pre-publication history for this paper can be accessed here:

http://www.biomedcentral.com/1471-2474/12/176/prepub
